# A computational simulation appraisal of banana lectin as a potential anti-SARS-CoV-2 candidate by targeting the receptor-binding domain

**DOI:** 10.1186/s43141-023-00569-8

**Published:** 2023-11-28

**Authors:** Sofia Safitri Hessel, Fenny Martha Dwivany, Ima Mulyama Zainuddin, Ketut Wikantika, Ismail Celik, Talha Bin Emran, Trina Ekawati Tallei

**Affiliations:** 1 School of Life Sciences and Technology, Institut Teknologi Bandung, Bandung, West Java 40132 Indonesia; 2 Department of Biosystems, KU Leuven, Willem de Croylaan 42 box 2455, B-3001 Leuven, Belgium; 3 Remote Sensing and Geographical Information Science Research Group, Faculty of Earth Science and Technology (FITB), Institut Teknologi Bandung, Bandung, West Java 40132 Indonesia; 4 Department of Pharmaceutical Chemistry, Faculty of Pharmacy, Erciyes University, 38039 Kayseri, Turkey; 5 Department of Pathology and Laboratory Medicine, Warren Alpert Medical School, Brown University, Providence, RI 02912 USA; 6 Department of Pharmacy, Faculty of Allied Health Sciences, Daffodil International University, Dhaka, 1207 Bangladesh; 7 Legorreta Cancer Center, Brown University, Providence, RI 02912 USA; 8 Department of Biology, Faculty of Mathematics and Natural Sciences, Sam Ratulangi University, Manado, North Sulawesi 95115 Indonesia

**Keywords:** Banana lectin, Antiviral, SARS-CoV-2, Molecular dynamics simulations

## Abstract

**Background:**

The ongoing concern surrounding coronavirus disease 2019 (COVID-19) primarily stems from continuous mutations in the genome of the severe acute respiratory syndrome coronavirus 2 (SARS-CoV-2), leading to the emergence of numerous variants. The receptor-binding domain (RBD) in the S1 subunit of the S protein of the virus plays a crucial role in recognizing the host’s angiotensin-converting enzyme 2 (hACE2) receptor and facilitating cell membrane fusion processes, making it a potential target for preventing viral entrance into cells. This research aimed to determine the potential of banana lectin (BanLec) proteins to inhibit SARS-CoV-2 attachment to host cells by interacting with RBD through computational modeling.

**Materials and methods:**

The BanLecs were selected through a sequence analysis process. Subsequently, the genes encoding BanLec proteins were retrieved from the Banana Genome Hub database. The FGENESH online tool was then employed to predict protein sequences, while web-based tools were utilized to assess the physicochemical properties, allergenicity, and toxicity of BanLecs. The RBDs of SARS-CoV-2 were modeled using the SWISS-MODEL in the following step. Molecular docking procedures were conducted with the aid of ClusPro 2.0 and HDOCK web servers. The three-dimensional structures of the docked complexes were visualized using PyMOL. Finally, molecular dynamics simulations were performed to investigate and validate the interactions of the complexes exhibiting the highest interactions, facilitating the simulation of their dynamic properties.

**Results:**

The BanLec proteins were successfully modeled based on the RNA sequences from two species of banana (*Musa* sp.). Moreover, an amino acid modification in the BanLec protein was made to reduce its mitogenicity. Theoretical allergenicity and toxicity predictions were conducted on the BanLecs, which suggested they were likely non-allergenic and contained no discernible toxic domains. Molecular docking analysis demonstrated that both altered and wild-type BanLecs exhibited strong affinity with the RBD of different SARS-CoV-2 variants. Further analysis of the molecular docking results showed that the BanLec proteins interacted with the active site of RBD, particularly the key amino acids residues responsible for RBD’s binding to hACE2. Molecular dynamics simulation indicated a stable interaction between the Omicron RBD and BanLec, maintaining a root-mean-square deviation (RMSD) of approximately 0.2 nm for a duration of up to 100 ns. The individual proteins also had stable structural conformations, and the complex demonstrated a favorable binding-free energy (BFE) value.

**Conclusions:**

These results confirm that the BanLec protein is a promising candidate for developing a potential therapeutic agent for combating COVID-19. Furthermore, the results suggest the possibility of BanLec as a broad-spectrum antiviral agent and highlight the need for further studies to examine the protein’s safety and effectiveness as a potent antiviral agent.

## Background

The coronavirus disease 2019 (COVID-19) pandemic, which has lasted 3 years, has infected 765,222,932 people worldwide and claimed 6,921,614 lives. Additionally, 13,344,670,055 doses of vaccine had been administered to mitigate the impact of the disease (https://covid19.who.int/; accessed on 3rd May 2023). Originating in Wuhan, China, COVID-19 was declared a pandemic by the World Health Organization on March 11, 2020. The disease is caused by the highly contagious severe acute respiratory syndrome coronavirus 2 (SARS-CoV-2), a novel betacoronavirus belonging to the Coronaviridae family. This family also includes other members, such as SARS-CoV and MERS-CoV [1].

The rapid mutation of SARS-CoV-2 has made it the most dangerous virus globally, with the emergence new viral variants. While many mutations in the SARS-CoV-2 genome are expected to be neutral, certain variations have been observed to alter viral function in terms of infectivity, illness severity, and host interactions [2]. Of particular concern are variants capable of evading antibodies, which can reduce the efficacy of some vaccines [3]. SARS-CoV-2 is more susceptible to mutations as it is an RNA virus, and mutations arise due to errors during RNA replication in the virus replication process. This results in the accumulation of mutated sequences and the emergence of diverse variants. The significantly higher error rates during replication may even confer a fitness advantage, leading to increased virulence of newly mutated viruses [4].

Various measures have been implemented to combat SARS-CoV-2 infection and its associated symptoms, such as restrictions, lockdowns, and improved general hygiene practices, and global vaccine developments and distribution. Vaccines have been effective in reducing the severity of infection and hospitalization rates [5]; however, their efficacy may wane over time following the second dose [6]. Moreover, the emergence of more contagious variants underscores the need for more sustainable and effective solutions. In addition to vaccination efforts, health professionals and researchers from diverse disciplines are working feverishly to develop an antidote for the disease.

Researchers are exploring repurposing antiviral drugs, such as lopinavir, ritonavir, nelfinavir, remdesivir, favipiravir, ribavirin, sofosbuvir, chloroquine, hydroxychloroquine, and azithromycin, to target key SARS-CoV-2 proteins and inhibit the virus [7, 8]. Ivermectin, an FDA-approved antiparasitic drug, has shown in vitro antiviral activity against SARS-CoV-2, but requires further research to confirm its efficacy and safety for COVID-19 treatment [9]. Gallinamide A and its analogues, derived from marine cyanobacteria, have demonstrated potent in vitro anti-SARS-CoV-2 activity by inhibiting cathepsin L, a host enzyme involved in viral entry [10]. Dual drug combinations, including antiviral agents, antibiotics, and hydroxychloroquine, have shown promising synergistic antiviral effects in vitro against SARS-CoV-2 isolated from hospitalized patients in Indonesia [11]. These studies emphasize ongoing efforts to develop effective antiviral medicines and phytochemicals to expand treatment options for COVID-19. Additionally, medicines derived from plants are also sought [12–16].

Molecular docking is a valuable in silico method used to predict potential interactions between molecules, allowing for the analysis of their properties and interactions, including proteins [17]. In the context of a pandemic, developing therapeutic agents for SARS-CoV-2 is challenging due to a lack of human resources and increased restrictions on public activities. This makes in silico methods of research, such as molecular docking, an appealing and interesting approach. As part of the search for new antivirals to combat COVID-19, researchers have been exploring carbohydrate-binding agents (CBAs) that target the N-linked glycans on the surface of the virus. Lectins are one class of CBAs that have shown promise in binding to viral glycoproteins and preventing virus transmission and penetration into host cells [18].

Bananas, one of the most commonly consumed fruits worldwide, have a plethora of health benefits [19, 20] and are easily accessible. Among the proteins found in ripe bananas is the banana lectin (BanLec), a dimeric protein composed of two 15-kDa subunits containing 141 amino acids each and is related to the jacalin lectin family [21]. BanLec is highly specific to mannose/glucose, which are viral cell surface glycans, making it an attractive candidate for antiviral development [22, 23]. Studies have shown that BanLec can inhibit HIV-1 reverse transcriptase activity [24], suppress influenza viral fusion [25], and provide protective activity against herpes simplex virus (HSV) type 1 [26]. Additionally, it has been observed to suppress cancer cell proliferation [27] and activate macrophages [28]. A mutation in BanLecs sugar binding site has been found to significantly reduce its mitogenic activity while maintaining its antiviral activity against viruses with high-mannose-type N-glycans on their surfaces, suggesting it has potential as a broad-spectrum antiviral agent [24]. Through a single mutation (Histidine to Threonine at position 84), the H84T BanLec demonstrated almost non-mitogenic properties while still retaining antiviral activity [29].

Despite extensive research and vaccination programs worldwide, the search for antiviral candidates against SARS-CoV-2 remains ongoing. Researchers are still exploring natural sources for potential antiviral medication. In this study, we investigated the potential of BanLec as anti-SARS-CoV-2 agent by examining its interaction with the RBD of wild-type, Delta plus, and Omicron variants.

## Materials and methods

### BanLec gene selection

To select BanLecs for this study, sequence analysis was conducted, with a focus on the GXXXD (that were found to be specifically GDXXD and GXFXD in the MSA analysis from Covés-Datson et al. [29]) motifs present in the two carbohydrate binding sites (CBSs) of BanLec protein sequences. Genes with these desired features were preferred for sequence and protein modification [29]. The genes encoding BanLec proteins were obtained from the Banana Genome Hub database (https://banana-genome-hub.southgreen.fr/; accessed on 21 April 2022). The protein sequence prediction was performed using the FGENESH online tool (http://www.softberry.com/berry.phtml?topic=fgenesh&group=programs&subgroup=gfind; accessed on 21 April 2022) [30]. The multiple sequence alignment (MSA) of the predicted protein sequences were performed using the MEGA 11 software [31], and the results were visualized with the UCSF Chimera package release 1.16 [32].

### Physicochemical properties, allergenicity, and toxicity prediction of BanLecs

The ProtParam web server (https://web.expasy.org/cgi-bin/protparam; accessed on 02 May 2022) [33] was utilized to calculate the theoretical physicochemical properties of the BanLec proteins. Theoretical allergenicity was analyzed using the Allergen FP v.1.0 web server (https://ddg-pharmfac.net/AllergenFP/; accessed on 02 May 2022) [34]. Prediction of toxicity and toxic domains was conducted using the ToxDL: Interpretable protein toxicity predictor web server (http://www.csbio.sjtu.edu.cn/bioinf/ToxDL/; accessed on 02 May 2022) [35].

### SARS-CoV-2 RBDs multiple sequence alignment and structural modeling

The wild-type RBD protein structure of SARS-CoV-2 was obtained from the RCSB Protein Data Bank with PDB ID 6M0J (https://www.rcsb.org/structure/6M0J; accessed on 07 May 2022). The RBD protein structures of the Delta and Omicron variants were modeled using the SWISS-MODEL web server (https://swissmodel.expasy.org/; accessed on 15 May 2022) [36–40], with wild-type RBD as a template. The sequences of the RBDs were modified through in silico mutagenesis according to the corresponding mutations (https://covdb.stanford.edu/page/mutation-viewer; accessed on 15 May 2022). The multiple sequence alignment (MSA) of the RBD sequences were performed using the MEGA 11 software [31], and the results were visualized with the UCSF Chimera package release 1.16 [32].

### Modeling of BanLecs three-dimensional structures

Modeling of BanLec's three-dimensional structures involved a selection process to identify two promising candidate protein for molecular docking. The design of *M. acuminata* BanLec protein utilized the selected sequence instead of readily available models from RCSB PDB in order to ensure uniformity and presence of the specific GDXXD and GXFXD motifs found in the CBS. In silico mutagenesis was then performed on the selected protein sequences to introduce a single amino acid mutation, specifically changing Histidine to Threonine at the 84th position in the *M. acuminata* BanLec protein and at the 115th position in the *M. balbisiana* BanLec protein. The three-dimensional (3D) structures of the proteins derived from the selected BanLec genes were modeled using the SWISS-MODEL web server (https://swissmodel.expasy.org/; accessed on 28 May 2022) [36–40]. To assess the accuracy of the protein modeling, Ramachandran plots were employed as a tool to evaluate the quality and stereochemical properties of the modeled proteins. By examining these plots, any deviations from the expected conformation were identified, offering valuable insights into potential errors that may have occurred during the modeling process [41, 42].

### Pre-docking preparation

Proteins modeled using SWISS-MODEL were subjected to pre-docking preparation, which involved the addition of hydrogen atoms and Kollman charges using the AutoDockTools software from MGLtools v.1.5.7 (https://ccsb.scripps.edu/mgltools; accessed on 05 July 2022) [43, 44]. Proteins downloaded from the PDB underwent water removal using AutoDockTools software, and ligand removal was carried out using the UCSF Chimera version 1.15 [32]. All of the proteins underwent energy reduction using the YASARA Energy Minimization Server (http://www.yasara.org/minimizationserver.htm; accessed on 05 July 2022) [45] and were converted into .sce format using YASARA View [46].

### Molecular docking analysis

The molecular docking utilized the ClusPro 2.0 protein-protein docking web server (https://cluspro.bu.edu/home.php; accessed on 10 July 2022) [47–49]. Calculation of the binding-free energy (ΔG) was conducted using PROtein binDIng enerGY prediction (PRODIGY) web server (https://wenmr.science.uu.nl/prodigy/; accessed on 10 July 2022) [50]. The interaction interfaces of the docked complexes were identified and analyzed through the EMBL-EBI PDBsum server (https://www.ebi.ac.uk/thornton-srv/databases/pdbsum/Generate.html/; accessed on 10 July 2022) [51]. Additional molecular docking analysis was performed using the HDOCK web server (http://hdock.phys.hust.edu.cn/; accessed on 14 July 2022) [52, 53] to obtain the docking scores for confirming the interaction of the complexes.

### Visualization of the best docked models

The ClusPro docking results were used to select the single best model of each complex. These complexes were then visualized in two dimension using the DIMPLOT program in LigPlot+ v.2.2 software [54]. The 3D structures of the docked complexes were visualized using PyMOL Molecular Graphics System software, version 2.0 [55].

### Molecular dynamic simulation study

The molecular dynamic simulation was performed following the protocols outlined by Celik et al. [41], utilizing the GROMACS 2019.2 version [56]. This simulation was conducted to investigate and confirm the interaction of the complexes and to simulate their dynamic properties. The topology created for the BanLec-RBD complexes utilized the AMBER99SB-ILDN force fields [57] and the SCP water model. The system was solvated in a triclinic box with 10Å distance from the protein–protein complex. The system was subsequently neutralized by adding 0.15 M NaCl, and the steepest descent integrator was used to perform energy minimization in 5000 steps. The equilibration of the system was accomplished with 0.3 ns NVT and 0.3 ns NPT stages, respectively, using a V-rescale thermostat and a Parrinello-Rahman barostat [58]. Simulation of 1000 frames for 100 ns was performed at 2 fs with a leap-frog integrator. Trajectory analysis was conducted using RMSD, RMSF, and Rg, and the plots were analyzed using QtGrace v0.2.6 [59].

**Fig. 1:**
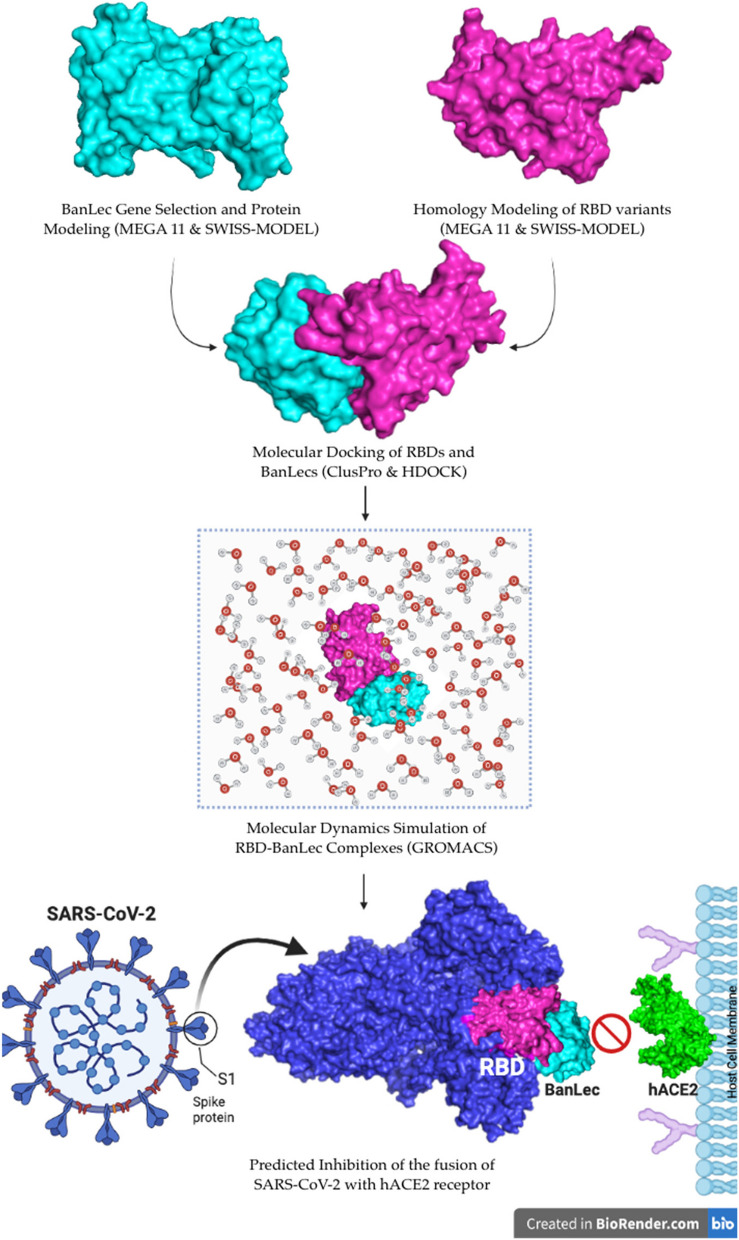
Visual representation of BanLec and SARS-CoV-2 RBD molecular docking and molecular dynamics simulation workflow (Created with BioRender.com)

The schematic representation of the study’s workflow and expected outcome can be seen in Fig. 1.

## Results

### BanLec gene selection

**Fig. 2:**
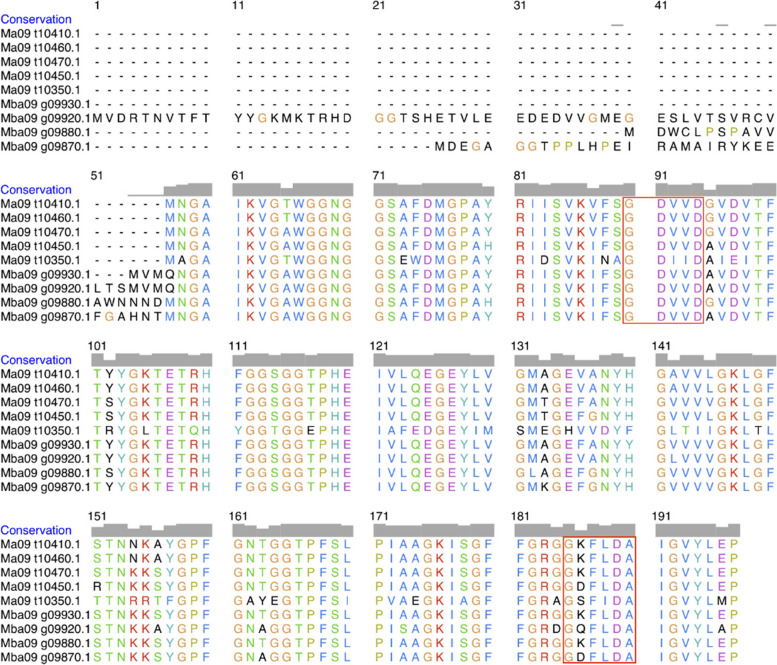
The MSA of nine BanLec protein sequences from *M. acuminata* and *M. balbisiana*, with the red boxes highlighting the two carbohydrate binding sites (CBS)

Nine BanLec candidates with the appropriate motifs from two *Musa* species (five from *M. acuminata* and four from *M. balbisiana*) were identified in our analysis. The MSA of the protein sequences and their motifs are highlighted (Fig. 2). Ma09_t10**0.1 is for *M. acuminata* BanLecs, and Mba09_g09**0.1 is for *M. balbisiana* BanLecs, with * representing any digit.

### Physicochemical property, allergenicity, and toxicity of BanLec

Table 1 shows the values of several physicochemical properties, allergenicity, and toxicity of the selected BanLec proteins (Ma09_t10410.1 WT, Ma09_t10410.1 H84T, Mba09_g09870.1 WT, and Mba09_g09870.1 H115T), where the BanLecs are seen to have an average molecular mass of 14,576.4 Da, an average theoretical isoelectric point of 6.16, and a computed average instability index of 8.97, where predicted allergenicity of the BanLecs resulted in an average Tanimoto index of 0.84, and an average ToxDL score of approximately 0.0268.

**Table 1:** Overview of the physicochemical property, allergenicity, and toxicity of the four selected BanLec proteins

BanLec protein	Molecule weight (kDa)	Theoretic pI	Instability index	Allergenicity	Toxicity
Ma09_t10410.1 WT	14,549.39	6.26	9.18	0.83	No toxic domains detected
Ma09_t10410.1 H84T	14,513.36	6.06	9.18	0.83	
Mba09_g09870.1 WT	14,639.52	6.26	9.44	0.83	
Mba09_g09870.1 H115T	14,603.48	6.06	8.09	0.84	

### MSA and homology modeling of RBD variants

**Fig. 3:**
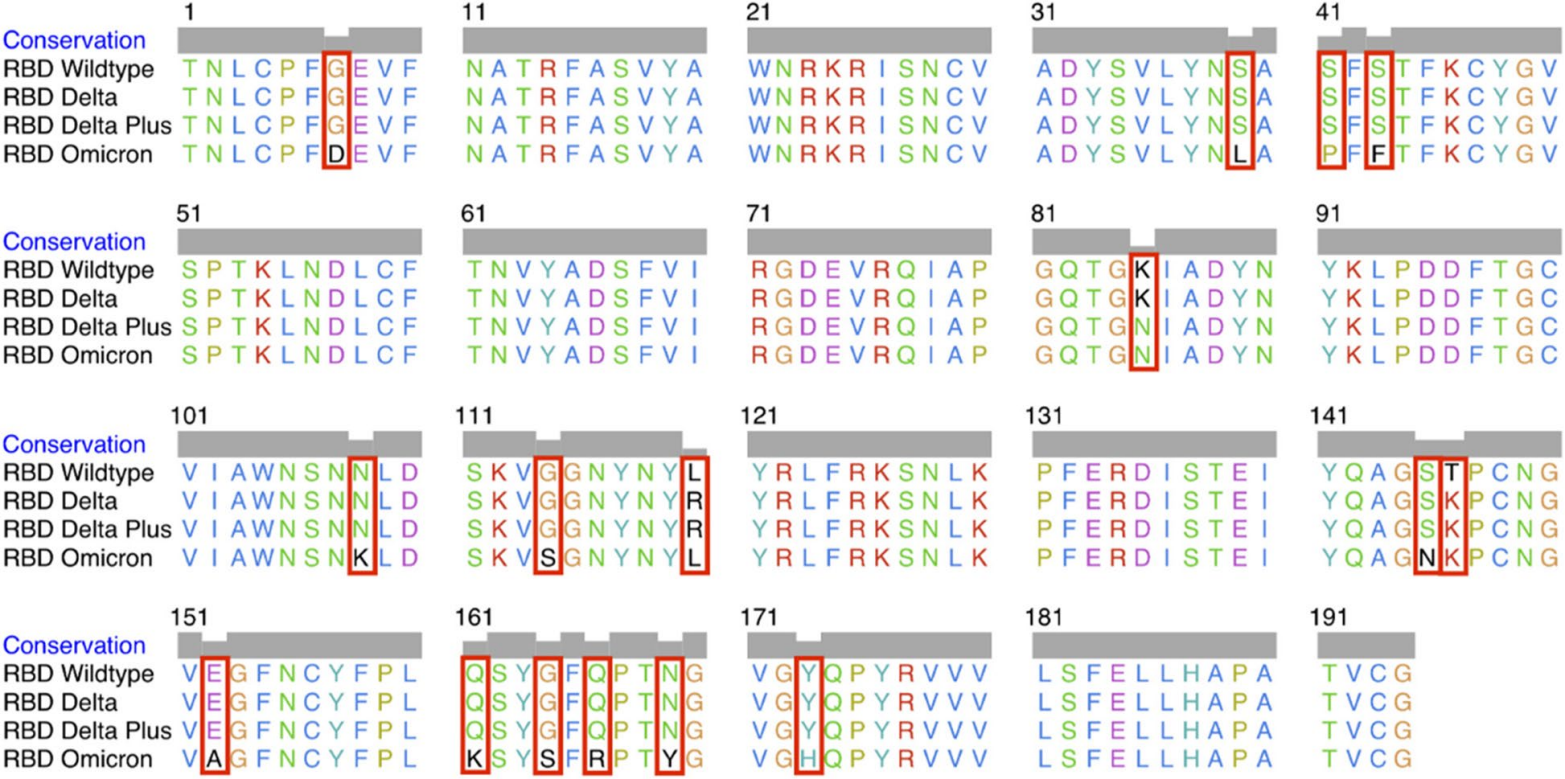
The MSA of four RBDs of SARS-CoV-2. The differences in amino acid sequences due to mutations are highlighted by red boxes

The MSA of the four RBD variants in this study confirmed the presence of mutations in the RBD variants, as depicted in Fig. 3 with mutations highlighted in red boxes. The WT-RBD protein was retrieved from the Protein Data Bank (PDB ID: 6M0J), chosen because molecular docking were successfully conducted using this structure in a previous research by Celik et al. [41]. The amino acid sequences of Delta and Delta Plus RBD proteins were also obtained from Celik et al. [41] .

**Fig. 4:**
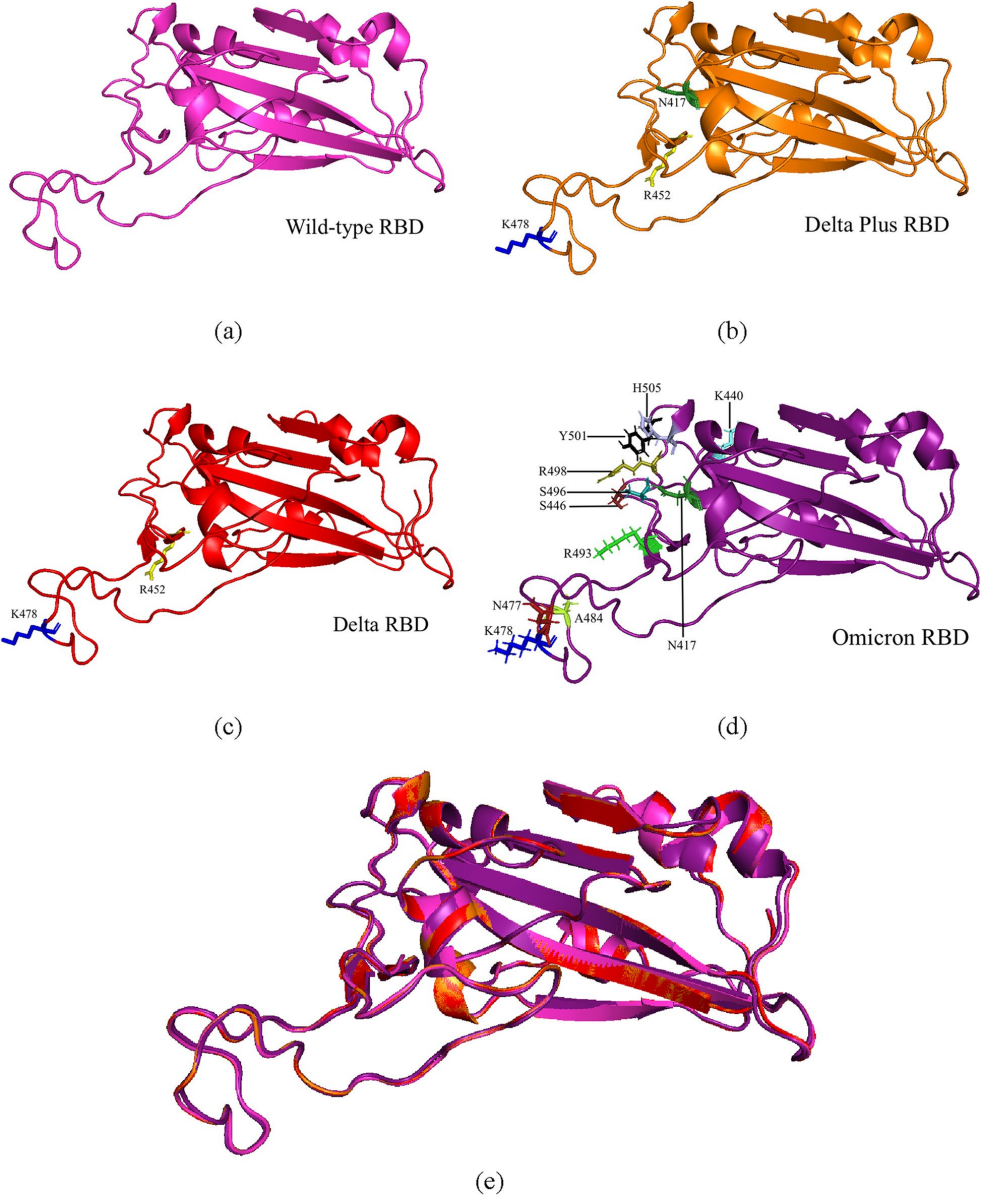
The three-dimensional (3D) structural models of four receptor-binding domains (RBDs) of SARS-CoV-2, which include the wild-type RBD and three variants (Delta, Delta Plus, and Omicron). **a** The wild-type RBD structure is represented by a ribbon visualization in a pink color. **b** The delta RBD structure is depicted as a ribbon in a striking red color, emphasizing its specific mutations (R452 and K478). **c** The delta plus RBD structure is presented as a ribbon in an orange color, with its mutations (N417, R452, and K478) highlighted in a vibrant yellow shade. **d** The Omicron RBD structure is showcased as a ribbon in a regal purple color, and its mutations (K440, S446, N417, N477, K478, A484, R493, S496, R498, Y501, and H505) are highlighted in a vibrant yellow color. **e** The superimposed model encompasses the comparative analysis of the structural conformation of various SARS-CoV-2 variants’ RBDs with the wild-type (WT) RBD

The amino acid sequences of the Omicron RBD protein were compiled based on this information. All proteins were modeled in SWISS-MODEL and visualized as ribbons, as shown in Fig. 4. The superimpose model involves comparing the structural conformation of various components, particularly the receptor-binding domain (RBD), with the wild-type (WT) RBD. In this case, the Delta RBD exhibits a root-mean-square deviation (RMSD) of 0.061Å compared to the WT-RBD, involving the alignment of 189 atoms. Similarly, the Delta Plus RBD shows an identical RMSD value of 0.061 Å, aligning 189 atoms with the WT-RBD. On the other hand, the Omicron RBD demonstrates a higher RMSD value of 0.304 Å, aligning 182 atoms with the WT-RBD.

### Homology modeling of BanLecs

After undergoing a selection process, the nine BanLec proteins were subjected to homology modeling via the SWISS-MODEL web server. Table 2 presents the names of gene sequences utilized for the protein modeling, with the “oligo state” denoting the type of protein assembly present in the modeled protein. The template specifies the PDB code for the template protein employed in the modeling process, while “the sequence’s identity” denotes percentage of similarity discovered in the target-template alignment. The global model quality estimate (GMQE) is a quality estimate derived from the combination of target-template alignment and template structure properties. The qualitative model energy analysis (QMEAN) is a composite scoring function that indicates global and local derivative of absolute quality estimates of a single model [60, 61].

**Table 2:** Detailed overview of the properties and description of the SWISS-MODEL homology modeling results of the nine modeled BanLec proteins

Gene name	Model	Oligo state	Template	Template description	Sequence’s identity	GMQE	QMEAN
Ma09_t10410.1	1	Homo-dimer	4pif.1.A	Ripening associated protein (Crystal structure of recombinant WT BanLec)	97.16%	0.92	2.20
Ma09_t10460.1	1	Homo-dimer	4pif.1.A	Ripening associated protein (Crystal structure of recombinant WT BanLec)	97.16%	0.92	2.20
Ma09_t10470.1	1	Homo-dimer	4pif.1.A	Ripening associated protein (Crystal structure of recombinant WT BanLec)	94.33%	0.91	2.52
Ma09_t10450.1	2	Homo-dimer	4pif.1.A	Ripening associated protein (Crystal structure of recombinant WT BanLec)	92.20%	0.90	2.44
Ma09_t10350.1	1	Homo-dimer	7kmu.1.A	Jacalin-type lectin domain-containing protein (Structure of WT Malaysian BanLec)	100.0%	0.89	2.51
Mba09_g09930.1	1	Homo-dimer	4pif.1.A	Ripening associated protein (Crystal structure of recombinant WT BanLec)	94.33%	0.89	2.25
Mba09_g09920.1	7	Homo-dimer	4pif.1.A	Ripening associated protein (Crystal structure of recombinant WT BanLec)	92.91%	0.57	2.21
Mba09_g09880.1	1	Homo-dimer	3miv.1.A	Lectin (Structure of Banana lectin-Glc-alpha(1,2)-Glc complex)	95.04%	0.76	1.50
Mba09_g09870.1	2	Homo-dimer	4pif.1.A	Ripening associated protein (Crystal structure of recombinant WT BanLec)	92.91%	0.70	2.67

**Fig. 5:**
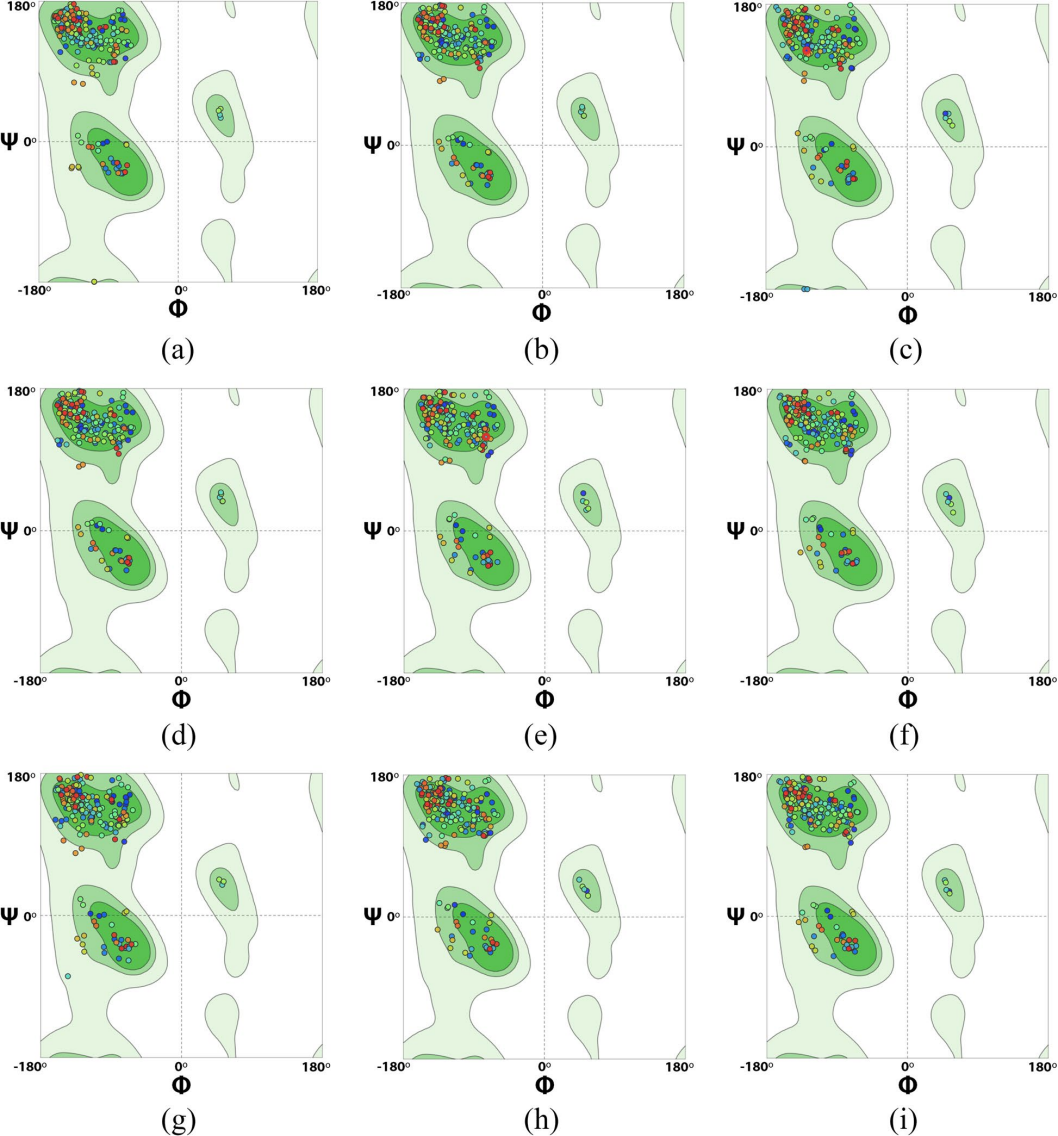
The Ramachandran plots obtained from the nine modelled BanLecs proteins derived from *M. acuminata* and *M. balbisiana*. The plots are presented individually for each protein: **a** Ma09_t10350.1, **b** Ma09_t10410.1, **c** Ma09_t10450.1, **d** Ma09_t10460.1, **e** Ma09_t10470.1, **f** Mba09_g09870.1, **g** Mba09_g09880.1, **h** Mba09_g09920.1, and **i** Mba09_g09930.1

The utilization of Ramachandran plots in protein modeling played a crucial role in assessing the precision of protein models, evaluating their accuracy, and identifying potential variations from the desired structural conformation. Figure 5 displays the Ramachandran plots for the nine modelled BanLecs proteins obtained from *M. acuminata* and *M. balbisiana*. These plots reveal that all amino acids reside predominantly in the favored regions, represented by dark green and light green contour lines. Only a few amino acids are located in the allowed regions, indicated by the lightest green contour line. The accompanying Table 3 further demonstrates that all models exhibit amino acid distribution within the Ramachandran favored regions ranging from 96.35 to 97.09%, with a maximum of 0.36% categorized as Ramachandran outliers. These results confirm the successful modeling, as the percentage coverage in the favored regions surpasses the 90% threshold indicative of high-quality models [42, 62].

**Table 3:** Distribution of amino acids in Ramachandran conformational regions in the nine modelled BanLec proteins

The modeled BanLec proteins	Ramachandran favored	Ramachandran outliers
Ma09_t10350.1	96.39%	0.00%
Ma09_t10410.1	97.09%	0.36% (B22 Pro)
Ma09_t10450.1	96.36%	0.36% (B22 Pro)
Ma09_t10460.1	97.09%	0.36% (B22 Pro)
Ma09_t10470.1	96.36%	0.36% (B22 Pro)
Mba09_g09870.1	96.36%	0.36% (B22 Pro)
Mba09_g09880.1	96.35%	0.00%
Mba09_g09920.1	96.73%	0.36% (B78 Pro)
Mba09_g09930.1	96.36%	0.36% (B25 Pro)

### Molecular docking of the BanLecs on WT-RBD

The initial molecular docking analysis involved nine modeled BanLec proteins and the WT-RBD. The analysis showed that while there were numerous interactions between the two proteins, there were not enough interactions with the key amino acid residues of the SARS-CoV-2 RBD. From the docked complexes, two models of each BanLec-WT-RBD complex were selected based on their lowest energy scores as determined by the ClusPro. The selected models were then further analyzed for their binding affinity using PRODIGY web server, which is a novel tool for predicting affinity [63]. Table 4 presents the analysis of the molecular docking results, including the lowest energy score, binding affinity, and interaction interface of the docked complexes.

**Table 4:** The properties and description of molecular docking results of the nine BanLecs with WT-RBD, which show the lowest energy score, binding affinity, and interaction interface

No.	Gene name	Criteria	Model	ClusPro lowest energy (LE)	Prodigy 𝚫G (kJ/mol)	Interaction interface (all residues)			Interaction interface (with key aa residues in RBD)		
						Salt bridges	H- bonds	Non-bonded	Salt bridges	H- bonds	Non-bonded
1.	Ma09_t10410.1	Best LE	Model 03	−1401.1	−16.1	3	27	260	-	-	-
		Best 𝚫G	Model 26	−1081.8	−17.4	1	26	297	-	4	1
2.	Ma09_t10460.1	Best LE	Model 03	−1401.1	−16.1	3	27	260	-	-	-
		Best 𝚫G	Model 26	−1081.8	−17.4	1	26	297	-	1	1
3.	Ma09_t10470.1	Best LE	Model 01	−1428.4	−16.0	1	29	253	-	-	14
		Best 𝚫G	Model 21	−1162.9	−16.3	1	26	260	-	-	-
4.	Ma09_t10450.1	Best LE	Model 05	−1272.0	−17.4	1	27	274	-	-	13
		Best 𝚫G	Model 05	−1272.0	−17.4	1	27	274	-	-	13
5.	Ma09_t10350.1	Best LE	Model 01	−1482.8	−14.3	3	18	224	-	-	-
		Best 𝚫G	Model 27	−1241.6	−18.7	3	22	244	-	-	-
6.	Mba09_g09930.1	Best LE	Model 02	−1441.3	−10.3	3	23	181	-	-	3
		Best 𝚫G	Model 21	−1137.4	−17.4	1	36	289	-	-	-
7.	Mba09_g09920.1	Best LE	Model 01	−1358.5	−11.7	2	22	204	-	-	3
		Best 𝚫G	Model 08	−1170.2	−18.3	4	27	322	-	-	-
8.	Mba09_g09880.1	Best LE	Model 03	−1296.0	−13.2	2	24	241	-	-	8
		Best 𝚫G	Model 21	−1050.3	−16.2	2	23	273	-	-	-
9.	Mba09_g09870.1	Best LE	Model 03	−1400.1	−16.0	-	31	245	-	1	14
		Best 𝚫G	Model 00	−1115.6	−17.1	2	16	515	-	-	-

Previous findings have identified specific amino acid residues in the RBD that are crucial for the interaction between the SARS-CoV-2 spike protein and hACE2. These amino acids are considered key amino acid residues, and their interactions are also observed in interactions in this study. The WT-RBD key amino acids include Lys417, Gly446, Tyr449, Tyr453, Leu455, Phe456, Ala475, Gly476, Phe486, Asn487, Tyr489, Phe490, Gln493, Gly496, Gln498, Thr500, Asn501, Gly502, and Tyr505 [64, 65]. Interestingly, the key residues considered in this study are the same for Delta, Delta plus, and Omicron variants, with variations depending on the position of the mutation. In the 6M0J protein structure, 10 hydrogen bonds and 1 salt bridge were identified between the hACE2 and WT-RBD protein. Specifically, a salt bridge formed with the Lys417 residue of WT-RBD, along with hydrogen bonds involving the Lys417, Gly446, Gly496, and Gly502 residues. Additionally, two hydrogen bonds were observed with each of the Tyr449, Asn487, and Thr500 residues [65].

**Fig. 6:**
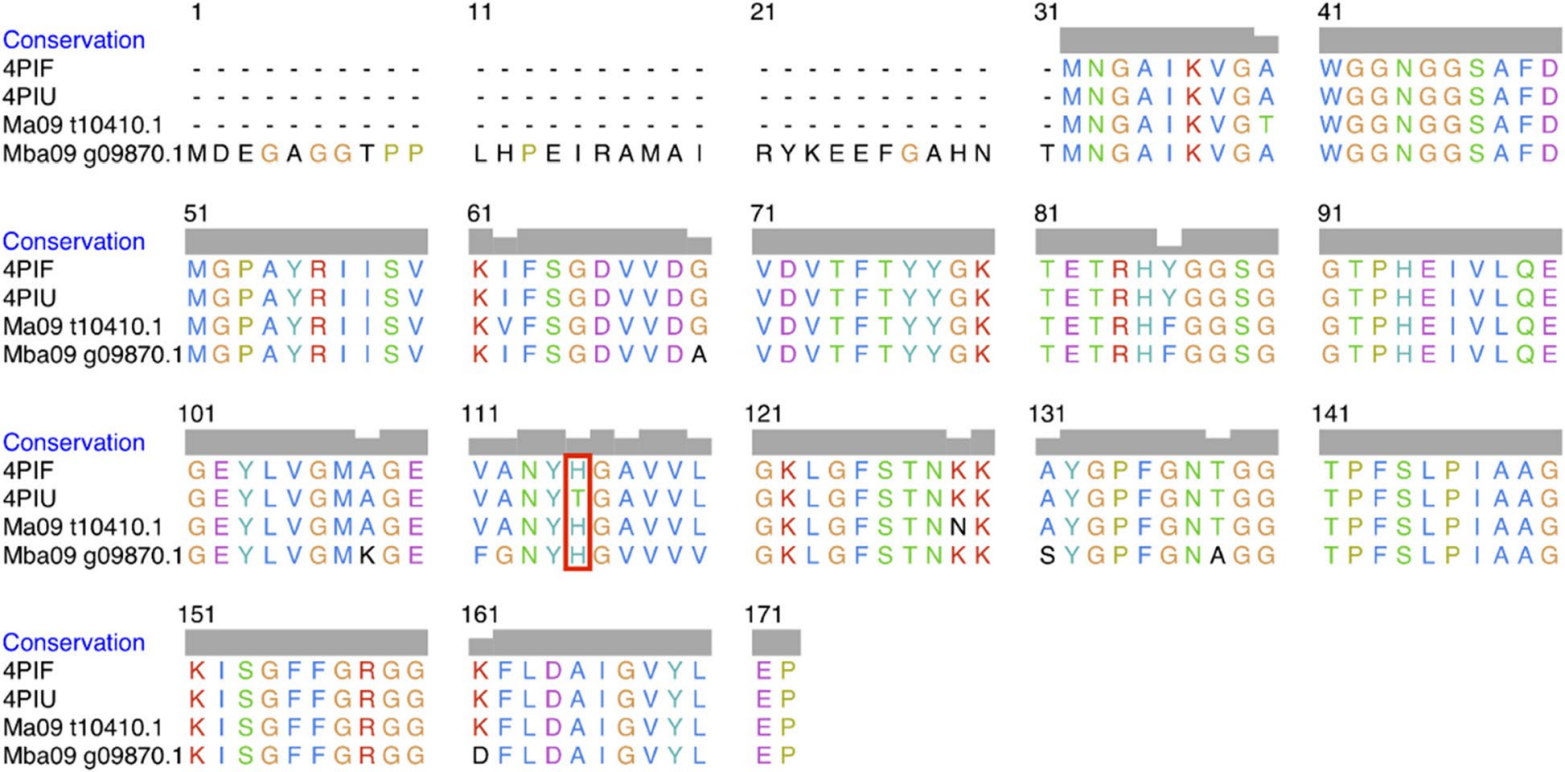
The MSA of four BanLec protein sequences derived from *M. acuminata* (Ma09_t10410.1) and *M. balbisiana* (Mba09_g09870.1). These protein sequences were aligned with both the reference sequence of WT (PDB ID: 4PIF) and the modified H84T *M. acuminata* BanLec (PDB ID: 4PIU). The amino acid sequence variations are visually emphasized by a red box

Out of the nine BanLecs considered, only two were chosen as the best candidates for in silico mutagenesis aimed at reducing their mitogenicity: Ma09_t10410.1 from *M. acuminata* (hereafter referred to as Ma09) and Mba09_g09870.1 from *M. balbisiana* (hereafter referred to as Mba09). The selection was based on their interactions with key amino acid residues on the SARS-CoV-2 RBD and the binding affinity of their models. In silico mutagenesis involved changing the specific amino acid, Histidine, to Threonine. The mutation was carried out at the 84th position for Ma09 and the 115th position for Mba09. The difference in sequence length between the two protein sequences cause different mutation positions. Instead of in the 84th position seen in the MSA, the mutation was carried out on the 115th position for Mba09. This different is attributed to the Mba09 sequence having an extra 31 residues in the beginning of the sequence, highlighted in the MSA in Fig. 6. However, the different positions in the aligned protein sequences do not change the actual position of the in silico mutagenesis carried out in the amino acids of the two sequences. As mentioned above, the mutation was performed at the 84th position for Ma09 and the 115th position for Mba09. The two protein sequences were also aligned with reference sequence of WT (PDB ID: 4PIF) and the modified H84T *M. acuminata* BanLec (PDB ID: 4PIU) from a previous study [24].

**Fig. 7:**
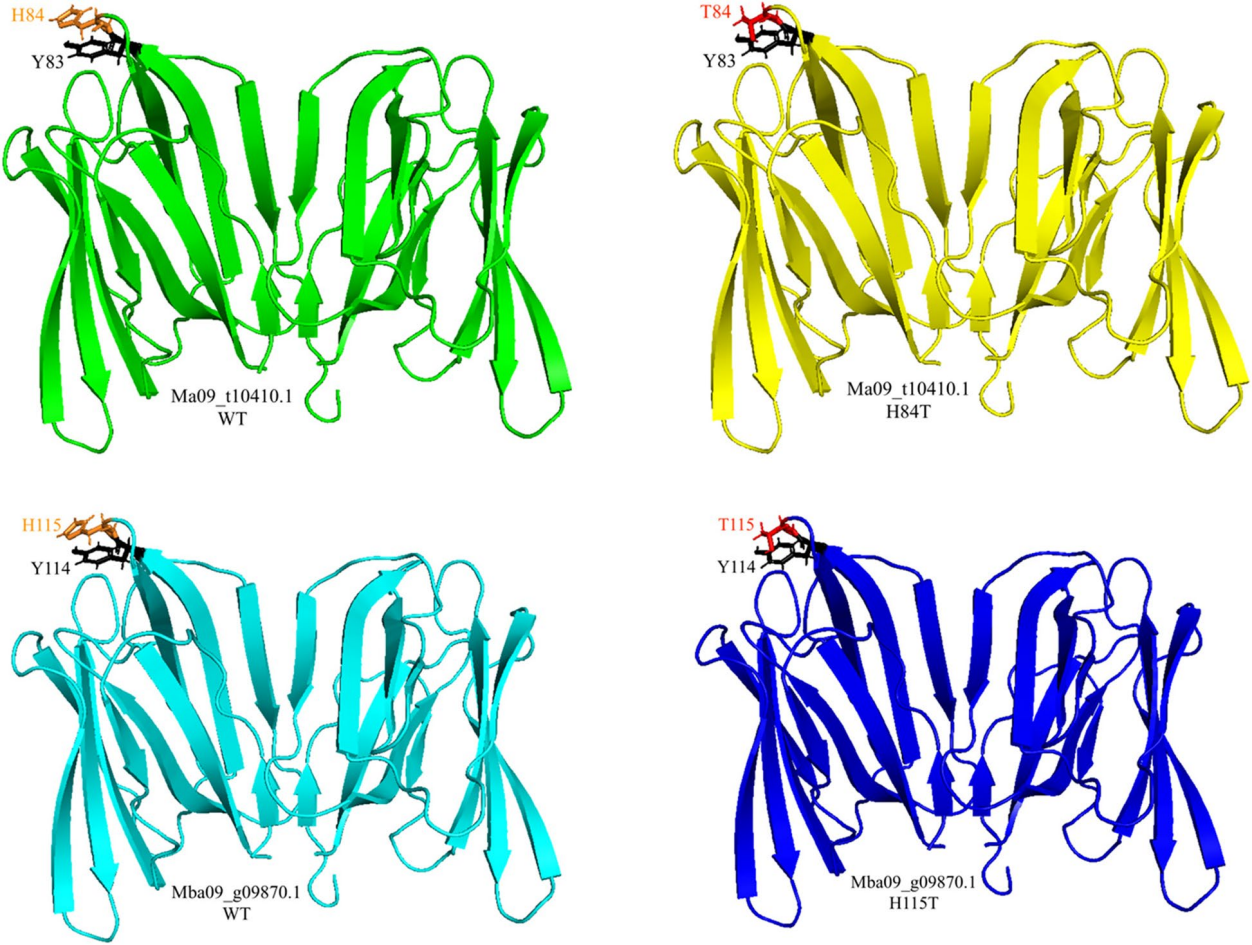
The 3D structural model of four BanLec proteins, with a specific mutation site highlighted. The mutation involved replacing Histidine 84/115 (H84/115) with Threonine 84/115 (T84/115), resulting in the disruption of a crucial pi-pi stacking interaction between H84/115 and Y83/114. Both these amino acids have aromatic rings, and pi-pi stacking refers to the non-covalent interactions that occur between these aromatic rings

The 3D structures of the four modeled BanLecs (two WT and 2 mutated BanLecs) were visualized using PyMOL and are shown in Fig. 7, where the mutated sites are highlighted. The loss of a pi-pi stack between Histidine (position 84 in Ma09 and 115 in Mba09) and Tyrosine (position 83 in Ma09 and 114 in Mba09), which are both aromatic rings, was observed as a result of the in silico mutagenesis of the Histidine (H84/115) into Threonine (position 84 in Ma09 and 115 in Mba09 or T84/115).

### Molecular docking of selected BanLecs on RBD

The interactions between protein-protein complexes were analyzed by docking the four variants of RBD and the four BanLec proteins. ClusPro 2.0 provides various scoring methods, in this study, the Balanced Scoring Coefficients [47] was utilized. The models with lowest energy were selected as the best docked models. Additionally, PRODIGY was used to analyze the binding affinity of each docked complex. Table 5 summarizes the binding properties of the best-selected complex from each molecular docking simulation. The best models were chosen based on their lowest energy and binding affinity predicted using ClusPro.

**Table 5:** The properties and description of the molecular docking results for the four SARS-CoV-2 RBD variants with the four selected BanLecs showing the lowest energy score, binding affinity, and interaction interface

SARS-CoV-2 variants	BanLec	Best model/cluster	ClusPro lowest energy (LE)	Prodigy 𝚫G (kJ/mol)	Interaction interface (all residues)			Interaction interface (with key aa residues in RBD)		
					Salt bridges	H- bonds	Non-bonded	Salt bridges	H- bonds	Non-bonded
WT	Ma09 WT	Model 26	−1081.8	−17.4	1	26	297	-	1	4
	Ma09 H84T	Model 01	−1219.9	−12.9	2	19	183	1	10	125
	Mba09 WT	Model 03	−1400.1	−16.0	-	3	245	-	1	14
	Mba09 H115T	Model 01	−1221.0	−14.9	2	25	214	1	19	124
Delta	Ma09 WT	Model 01	−1322.6	−10.7	-	9	125	-	4	81
	Ma09 H84T	Model 19	−1149.9	−14.8	2	19	250	1	14	200
	Mba09 WT	Model 14	−1149.5	−16.1	2	15	220	1	8	171
	Mba09 H115T	Model 04	−1303.5	−13.7	2	12	162	-	6	88
Delta Plus	Ma09 WT	Model 22	−1113.7	−16.3	-	17	218	-	11	166
	Ma09 H84T	Model 12	−1173.5	−16.8	-	-	-	3	-	-
	Mba09 WT	Model 29	−1107.5	−16.4	-	12	179	-	12	179
	Mba09 H115T	Model 01	−1311.6	−12.8	3	14	187	-	9	133
Omicron	Ma09 WT	Model 20	−1208.9	−14.7	3	22	278	2	14	213
	Ma09 H84T	Model 00	−1376.5	−10.8	1	15	166	1	13	140
	Mba09 WT	Model 05	−1370.4	−15.2	2	17	209	-	7	125
	Mba09 H115T	Model 00	−1427.4	−14.4	3	15	217	-	8	162

The interaction between BanLecs and WT-RBD was characterized by a higher number of salt bridges, H-bonds, and non-bonded interactions, with a greater total of H-bonds and non-bonded interactions with all residues than the other variants. BanLecs however showed a preference for the Omicron RBD when considering the RBD’s key amino acid residues. This predilection was due to the presence of more H-bonds, non-bonded interactions, and salt bridge interactions within the key residues compared to the other variants. In addition to the overall proclivity for BanLecs, each RBD variant also exhibited a preference for specific BanLecs based on the interactions and bonds within the key RBD residues.

The selection of the best modeled complex of each RBD with its corresponding BanLec protein took into account the binding affinity and the total number of interactions and bonds between the two proteins. To confirm the interaction between the RBD variants and their corresponding BanLec protein, additional molecular docking was performed using the HDOCK web server (Table 6). A two-step docking strategy using ClusPro and HDOCK provides multiple benefits for predicting protein-protein complexes. These methods employ different algorithms and scoring functions, leading to diverse perspectives that can potentially improve accuracy. The strategy involves an initial round with ClusPro to explore a wide range of docking poses, followed by a second round with HDOCK to refine and optimize the solutions obtained from ClusPro. By combining these programs, limitations specific to each method can be overcome, and challenges in certain protein-protein interactions can be addressed. The consistency of predictions from both ClusPro and HDOCK serves as validation and increases confidence in the accuracy of the docking results. However, it is essential to critically evaluate the outcomes and consider the unique characteristics of the target proteins and the specific docking problem at hand.

**Table 6:** HDOCK molecular docking results of the four best RBD-BanLec complexes showing the docking score and ligand RMSD

SARS-CoV-2 variants	BanLecs	Docking score (kJ/mol)	Ligand RMSD (Å)
WT	Mba09 H115T	−270.71	77.70
Delta	Ma09 H84T	−277.70	67.11
Delta Plus	Mba09 WT	−249.66	70.56
Omicron	Ma09 WT	−290.10	58.49

The HDOCK scores were calculated using binding affinity predictions and provide insight into the quality of the models created and the accuracy of the homology-modeled structure [53]. Among the four complexes, the Omicron-Ma09 WT complex yields the best docking score (−290.10 kJ/mol) with the ligand RMSD of 58.49Å. While the scores are not vastly distinct, this affirms that Omicron RBD has a predilection for BanLec as compared to the other variants.

**Fig. 8:**
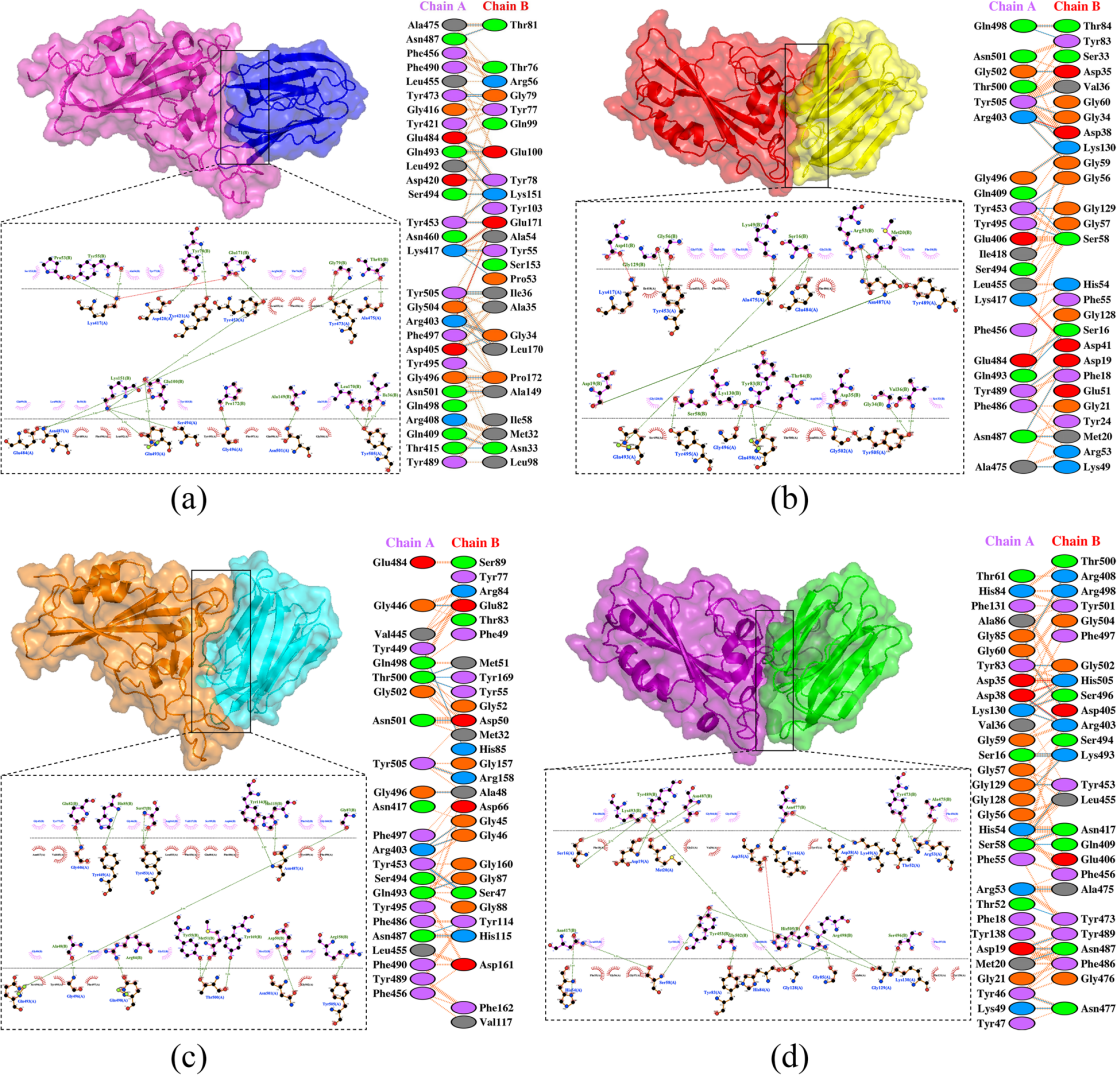
The diagrammatic representation in 3D and 2D configurations of the complex interface. **a** Wild-type RBD-Mba09 H115T complex (Chain A: RBD; Chain B: BanLec), **b** Delta RBD-Ma09 H84T complex (Chain A: RBD; Chain B: BanLec), **c** Delta plus RBD-Mba09 WT complex (Chain A: RBD; Chain B: BanLec), and **d** Omicron RBD-Ma09 WT complex (Chain A: BanLec; Chain B: RBD). In the PDBsum graphics (shown on the right), hydrogen bonds are indicated by blue straight lines, non-bonded contacts are represented by orange dashed lines, and salt bridges are denoted by red straight lines. In the LigPlot graphics (shown below the 3D graph), the representation of the ligands and protein side chains is depicted in a ball-and-stick format. The ligand bonds are shown in purple, while hydrogen bonds are depicted as green dotted lines with their length printed in the middle. Spoked arcs indicate protein residues that have non-bonded contacts with the ligand. Additionally, red circles and ellipses highlight protein residues that occupy similar positions in 3D when the two structural models are aligned

Figure 8 illustrates the 3D and 2D configurations of the docking results of the four complexes. The interface interaction of the four complexes was further assessed using PDBsum. The Omicron RBD-Ma09 WT complex displays the highest number of interactions with 213 non-bonded interactions, 14 H-bonds, and two salt bridges. The Delta RBD-Ma09 H84T complex contains 200 non-bonded interactions, 14 H-bonds, and one salt bridge. Delta plus RBD-Mba09 WT complex has 179 non-bonded interactions, 12 H-bonds, and no salt bridges. Lastly, the WT-RBD-Mba09 H115T complex has 124 non-bonded interactions, 19 H-bonds, and one salt bridge.

Figure 8a illustrates the interactions between the modified H115T *M. balbisiana* BanLec and the wild-type SARS-CoV-2 RBD. These interactions involve the formation of a salt bridge with Lys417 and H-bonds with Lys417, Tyr453, Ala475, Asn487, Gln493, Gln496, Gln498, Asn501, and Tyr505, which are key amino acid residues in the wild-type RBD. Figure 8b displays the interactions between the modified H84T *M. acuminata* BanLec and the Delta variant RBD. The interactions observed in this case include H-bonds and salt bridges formed between the BanLec and important amino acids in the RBD, such as a salt bridge with Lys417, and H-bonds with Tyr453, Ala475, Asn487, Tyr489, Gln493, Gly496, Gln498, Gly502, and Tyr505. Figure 8c demonstrates the interactions between the wild-type *M. balbisiana* BanLec and the Delta Plus variant RBD. In this case, H-bonds are formed between the BanLec and key amino acids in the RBD, specifically at residues Tyr449, Tyr453, Asn487, Gln493, Gln496, Thr500, Asn501, and Tyr505. Figure 8d presents the interaction between the wild-type *M. acuminata* BanLec and the Omicron variant RBD. The interactions observed involve both H-bonds and salt bridges formed between the BanLec and crucial amino acids in the RBD. The salt bridge occurs at residue His505, and H-bonds occur at residues Asn417, Tyr453, Ala475, Asn487, Tyr489, Arg493, Ser496, Gly502, and His505.

### Molecular dynamics simulations

**Fig. 9:**
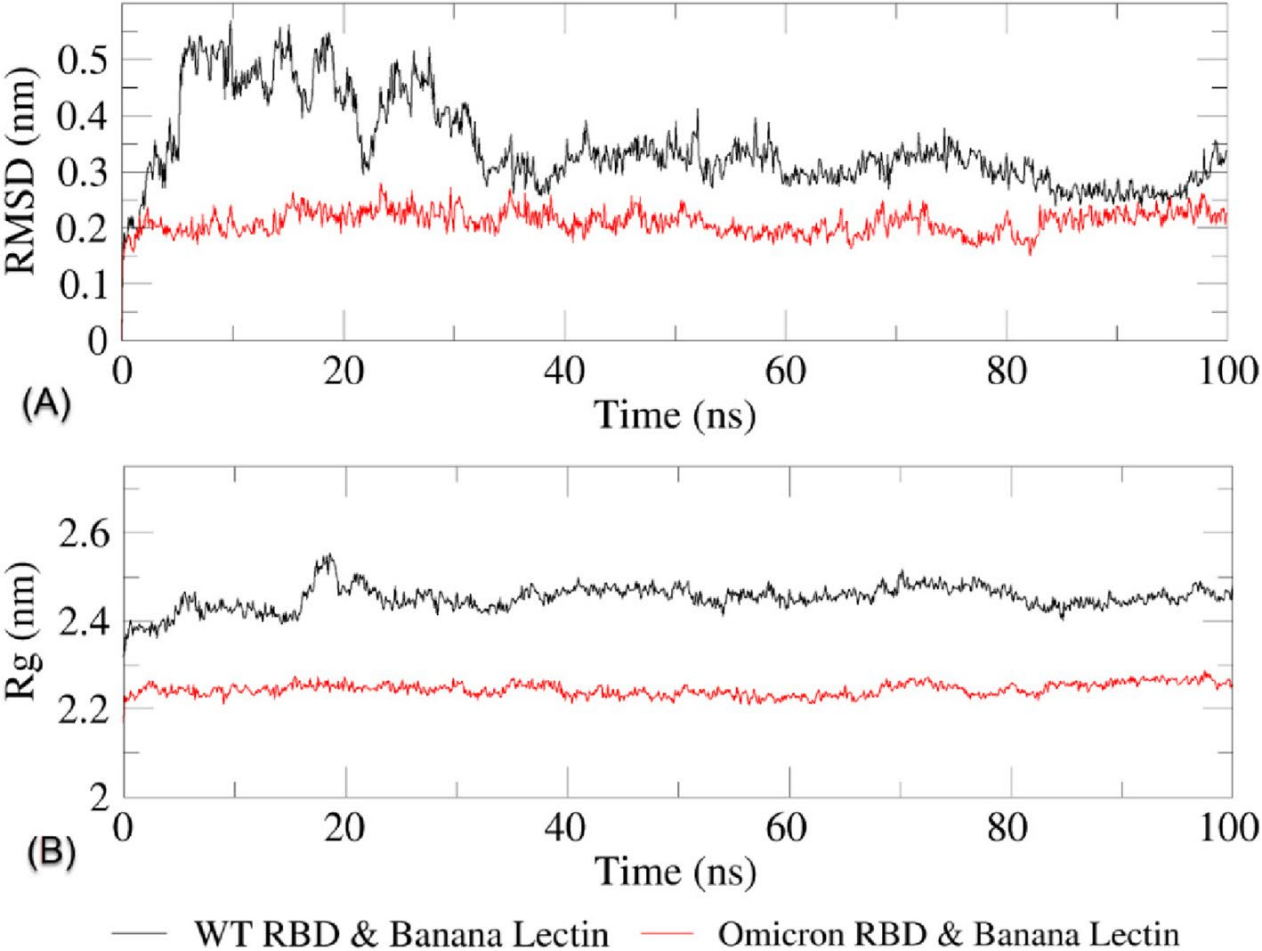
**A** RMSD and **B** Rg of SARS-CoV-2 WT and Omicron variants RBD complexed with their corresponding banana lectins

Molecular dynamics simulations (MDS) was performed on the WT-RBD-Mba09 H115T and Omicron RBD-Ma09 WT complexes based on their binding affinity, number of salt bridges, hydrogen bond, and non-bonded interaction with key RBD amino acid residues. The results of the RMSD and Rg analyses, which indicate a significant difference in overall stability between the two complexes, are illustrated in Fig. 9. The overall stability of the RBD-BanLec complexes was demonstrated using the primary molecular dynamics (MD) parameters of root-mean-square deviation (RMSD), radius of gyration (Rg), and root-mean-square fluctuation (RMSF).

In the case of the WT-RBD-Mba09 H115T complex, the RMSD shows more fluctuation, particularly in the first 40 ns, ranging from 0.25 to >0.5 nm, then decreases to a steadier level. However, a fluctuation at 0.25 and 0.4 nm is observed between 40 and 100 ns, and the complex tends to dissociate in the end of the simulation. On the other hand, the Omicron RBD-Ma09 WT complex exhibits greater stability throughout the 100 ns simulation, with the RMSD beginning at 0.15 nm and never falling below or exceeding 0.3 nm. The Rg value of the WT-RBD-Mba09 H115T complex is much more stable than its RMSD value, ranging from 2.35 to 2.55 nm overall throughout 0–100 ns, with a fluctuation up to 2.5 nm at around 18 ns. However, it is still higher and less steady than the Rg value of the Omicron RBD-Ma09 WT complex, which remains stable around 2.25 nm from 0 to 100 ns.

**Fig. 10:**
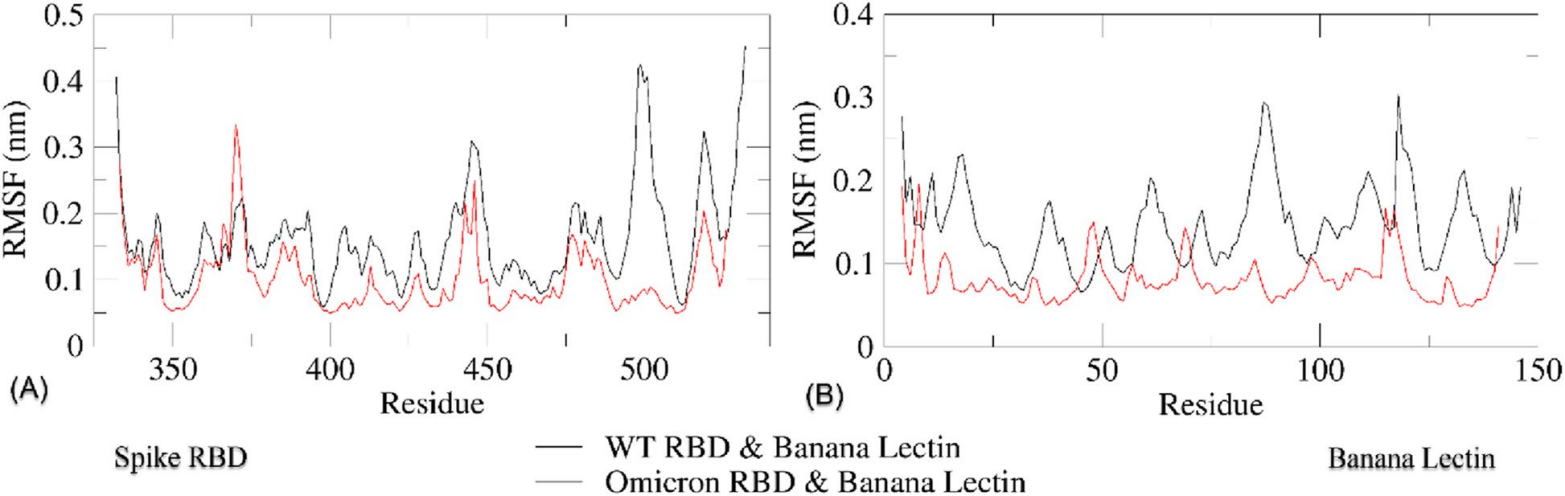
Residual RMSF of **A** SARS-CoV-2 RBD variants and **B** their corresponding banana lectin

The analysis results of the residual RMSF of each complex are shown in Fig. 10. The RMSF values for WT-RBD range from 0.07 to 0.45 nm, while those for Omicron RBD range from 0.05 to 0.35 nm, indicating slightly higher values and larger fluctuations in the graph. The counterpart of Omicron RBD, Ma09 WT BanLec displays a more stable structure with RMSF values ranging from 0.05 to 0.2 nm across all residues, while the WT-RBD counterpart, Mba09 H115T BanLec, exhibits greater fluctuation with values ranging from 0.08 to 0.35 nm

Table 7 shows the results of the MM-PBSA binding-free energy (BFE) calculation conducted on the 100 ns MDS with 1000 frames. A notable difference was observed in the calculated BFE values between the Omicron RBD-Ma09 WT complex (−209.67±34.14 kJ/mol) and the Wild-type RBD-Mba09 H115T complex (−17.66±51.44 kJ/mol), showing a lower BFE value for the Omicron RBD-Ma09 WT complex. Lower BFE values indicate a more stable complex.

**Table 7:** The BFE values generated between the interaction of SARS-CoV-2 spike proteins and BanLec as calculated using MM-PBSA

Protein complex	Types of interaction energy (kJ/mol)				
	Van der Waals	Electrostatic	Polar solvation	SASA	Total binding-free energy
Omicron RBD- BanLec	−378.958±30.662	−556.282±52.801	773.583±58.558	−48.015±3.089	**−209.672±34.141**
WT-RBD-BanLec	−283.760±29.761	−259.071±55.518	564.757±89.688	−39.585±3.685	**−17.658±51.442**

## Discussion

BanLec genes are abundant in several species of banana (*Musa* spp.). However, the genome of one banana species only contains a few variations of lectin-coding genes, making it necessary to select the best candidate for the development of BanLec protein as a potential antiviral. BanLec genes with a specific motif in their ligand binding loop sites, which characterizes a carbohydrate binding site, are preferred sequences for protein sequence and structure modification [29]. The presence of this specific motif gives the sequences their preferred structural properties, which include two carbohydrate binding sites (CBS), instead of having only one or zero CBS in their structure. These sites may contribute to stronger interactions and binding between BanLec and mannose/glucose. The presence of the second CBS was revealed in a study by Meagher et al. [21], and it has similar properties to the first CBS.

The selected BanLec proteins (Ma09_t10410.1 WT, Ma09_t10410.1 H84T, Mba09_g09870.1 WT, and Mba09_g09870.1 H115T) have an average molecular mass of 14,576.4 Da, a theoretical isoelectric point of 6.16, and a computed instability index of 8.97, classifying the proteins as stable. The predicted allergenicity of the BanLecs resulted in an average Tanimoto index of 0.84, signifying a high similarity between the two sets of protein fingerprint in the allergenicity analysis and suggesting that the BanLecs are probably non-allergenic. Allergenicity refers to the capability of a molecule or compound to induce a Th2 response and production of IgE antibodies by B cells that are allergen-related [66, 67]. One study showed no evidence of type I allergy to BanLec, as demonstrated by the lack of BanLec-specific serum IgE [68].

Toxicity of molecules or compounds can be calculated in silico by analyzing amino acid sequences and identifying toxic domains. According to toxicity prediction, the BanLecs have an average ToxDL score of approximately 0.0268, which is considered to be low toxic. The higher the score, the less likely the protein will be toxic. This score was calculated by adding the scores assigned to individual amino acids, where some amino acids contributed more to toxicity than others [35]. Despite the indications of low toxicity, the analysis reveals that none of the BanLec proteins contain toxic domains. Essentially, BanLec has been shown to have mitogenic activity [69]. However, it is still possible to engineer this protein produce BanLec that retains its efficacy with minimal or no mitogenic activity [70]. The values above are estimates and predictions based on in silico simulations, necessitating additional in vitro testing prior to implementing BanLecs as a therapeutic agent.

SARS-CoV-2, with its single-stranded RNA genome, is susceptible to rapid and higher rates of mutation [71], which may lead to changes in viral fitness, including improved infectivity, illness severity in the host, and other viral functions [2]. While mutations can occur anywhere along the viral genome, this study highlights mutation in RBD of the SARS-CoV-2 spike subunit 1 protein. The wild-type (WT) RBD (Wuhan-Hu-1; NCBI ID: P0DTC2) was used as a reference, with the Delta (B.1.617.2; NCBI ID: QWK65230.1) RBD containing two mutations, L452R and T478K, and the Delta Plus containing three mutations, K417N, L452R, and T478K. Omicron (B.1.1.529), the most recent variant of concern (VOC), has more mutations than the previously predominant Delta variant, including K417N, N440K, G446S, S477N, T478K, E484A, Q493R, G496S, Q498R, N501Y, and Y505H [72]. The presence of all of these mutations were confirmed in an MSA shown in Fig. 3. The WT-RBD and its variants were modelled and superimposed in this study. The RMSD values of the superimposed RBD protein structures seen in Fig. 4 provide insights into the structural differences and similarities between the RBD variants and the WT-RBD, highlighting the extent of their deviations at the atomic level. A higher RMSD value signifies a more pronounced distinction or deviation between a protein structure and its wild-type reference. This indicates substantial alterations or mutations in the protein structure, which can potentially impact its function and overall configuration.

According to Covés-Datson et al. [29], Swanson et al. [24], and the findings in our studies, there is a pi-pi stack in the BanLecs between a Histidine (position 84 in Ma09 and 115 in Mba09) and a Tyrosine (position 83 in Ma09 and 114 in Mba09), which are both aromatic rings. Pi-pi stacking refers to the attractive, non-covalent interactions between aromatic rings. This disruption of the pi-pi stack has been linked to a reduction in mitogenicity while preserving broad-spectrum antiviral properties [29]. This may be due to the ability of the protein to retain wild-type conformational features and could also be related to properties of the Threonine 84/115 side chain [24, 29]. The pi-pi stacking is diminished by the in silico mutagenesis since T84/115 does not have an aromatic ring structure, unlike H84/115.

The results of the molecular docking simulation of the four BanLecs (two WT and two mutated) with the RBD variants (Table 5) exhibited that at the critical or key amino acids, BanLecs interacted strongly with each RBD variant. The presence of a greater the number of bonds and interactions in a docked complex signifies a better prediction of binding and confirms the ability of BanLecs to establish interactions with the RBD of the SARS-CoV-2 spike S1 protein. H-bonds are covalent bond formed between a hydrogen atom and an extremely electronegative atom, and they are considered strong when they occur in large or multiple numbers [73]. A salt bridge is an ion pair that forms between two side chains of a protein. Even though it is comprised of non-covalent bonds, it significantly contributes to protein stability and overall protein binding [74]. Salt bridges and H-bonds interaction have been shown to play a critical role in protein-ligand stability [75]. The strength of H-bonds has been correlated to the distance or length between the two molecules, where shorter distances signify a stronger bond [76], and as seen from the green dotted lines in Fig. 8, the length hydrogen bonds of all complexes range from 2.00 to 3.36 Å. According to McRee [77], the distance of hydrogen bonds is commonly from 2.7 to 3.3 Å, with 3.0 Å being the most common value. This demonstrates that the H-bond strengths of the four best complexes range from moderate to strong.

In terms of the number of interactions and bonds, the RBD of SARS-CoV-2 exhibits a slightly higher preference for *M. acuminata* BanLecs. However, it shows a higher binding affinity value in its interactions with *M. balbisiana* BanLecs. In another stage of molecular docking, it was observed that BanLec Ma09 WT exhibits a stronger affinity towards Omicron RBD, suggesting its potential to hinder RBD binding to hACE2. Nevertheless, the ligand RMSD values in the molecular docking results for the four top BanLec-RBD complexes ranged from 58.49 to 77.70 Å. In contrast, the ligand RMSD values for hACE2 docked with RBD variants in the study by Celik et al. [41] ranged from 0.34 to 0.61 Å. A more negative ligand RMSD value indicates less deviation from its reference position, hence a better docking result. It is important to note that hACE2, being the natural receptor molecule for the SARS-CoV-2 RBD, is larger in size (603 aa in 6M0J PBD structure) compared to BanLecs (140–171 aa) [65], which may contribute to their better RMSD values when docked with the RBDs. Nonetheless, the analyzed binding affinity values of each BanLec-RBD complex (rangin from −14.7 to −16.4 kJ/mol) still show a more negative value compared to results of binding affinity between docked hACE2 and RBD variants in the study by Celik et al. [41], which ranged from −12.8 to −14.2 kJ/mol.

These results suggest that BanLecs are capable of binding to the active site of RBDs from various variants with high affinity. Moreover, they can bind to key amino acids in the RBDs that play a crucial role in RBD's interaction with hACE2. Therefore, these findings indicate that BanLecs have the potential to effectively inhibit the early stages of SARS-CoV-2 infection in human cells. This aligns with previous reports indicating that certain lectins derived from natural sources can bind to glycans on viral glycoproteins, thereby preventing virus transmission and entry into host cells [70, 78–80].

The stability of interactions is a crucial aspect to consider when analyzing molecular interaction predictions, as in the case of this study. Molecular dynamics (MD) simulation is a computational chemistry technique that simulates the behavior of atoms and/or molecules over a specified time period. This provides visual representation of the molecular movement of the complex and its interactions. In other words, this method allows for the simulation of molecules’ time-dependent motion and enables exploration of their conformational space [81]. The backbone atoms of the complexes are used to calculate the RMSD, which was utilized to observe trajectory equilibration. Protein structure shifts and deviations can be determined by this important stability analysis parameter. Rg is also important because it calculates the distance between mass-weighted RMS values of atoms from their center of mass and provides information about the compactness and overall dimensions of proteins [64, 82, 83]. Trajectory results with lower and more constant RMSD and Rg values indicate a more stable complex, whereas higher and more fluctuating values indicate a more unstable complex. Results of the molecular dynamics simulation showed that both WT-RBD-Mba09 H115T and Omicron RBD-Ma09 WT complexes were fairly stable throughout the simulation, with the latter complex being more stable with lower and less fluctuating RMSD and Rg.

RMSF is a parameter used to measure the flexibility and/or mobility of protein structures by tracking their conformational changes over time [64, 82]. A lower RMSF value implies a less adaptable and mobile structure, while a higher value suggests the opposite. The residual RMSF trajectories offer insight into the fluctuations that visualize the conformational changes and flexibility of each of the proteins in a complex. The graph in Fig. 10 reveals that the RBDs of WT and Omicron undergo greater changes than their BanLec counterparts. Nevertheless, the Omicron RBD exhibits lower fluctuation than the WT-RBD, suggesting greater conformational stability. Higher RMSF values indicate greater protein flexibility, which may result in reduced stability due to increased susceptibility to conformational changes. The greater residual flexibility observed in the WT-RBD-Mba09 H115T complex compared to the Omicron RBD-Ma09 WT complex implies a higher level of mobility and flexibility, thus less stability. BFE values were observed to favor the stability of the Omicron RBD-Ma09 WT complex. According to RMSD, Rg, RMSF, and BFE values, the interaction between WT-RBD and the corresponding BanLec Mba09 H115T was found to be less stable, with both the RBD and BanLec proteins exhibiting higher levels of fluctuation and flexibility compared to the corresponding proteins in the Omicron RBD-Ma09 WT complex. These findings support the results of the molecular docking analysis, suggesting that the BanLec has a stronger preference for the Omicron RBD over the WT-RBD. These findings may contribute to explaining why the Omicron variant is more infectious than the WT SARS-CoV-2. The study results also suggest that the preference for BanLecs is stronger in Omicron RBD compared to WT-RBD, and this preference may extend to the hACE2 protein.

## Conclusions

This study was conducted to explore the potential of BanLec proteins as a potent antiviral candidate against SARS-CoV-2 by simulating their binding with four variants of SARS-CoV-2 RBDs. Molecular docking analysis revealed strong interactions and bonds in the active site between the SARS-CoV-2 RBD variants and different BanLec proteins from *M. acuminata* and *M. balbisiana*. Moreover, molecular dynamics simulation demonstrated that the Omicron variant RBD exhibited a stable and robust interaction with wild-type *M. acuminata* BanLec, providing further insight into complex stability. These findings suggest that BanLecs could serve as a potential antiviral agent against SARS-CoV-2 by inhibiting the fusion of the virus with host cell. However, further investigation is required to assess the safety and efficacy of BanLecs as antiviral agents.

## Data Availability

The data that support the findings of this study are available from the corresponding author upon reasonable request.

## Abbreviations

3D: Three-dimensional
Å: Ångström
BanLec: Banana lectin
BFE: Binding-free energy
CBS: Carbohydrate binding site
COVID-19: Coronavirus disease 2019
DIMPLOT: Dimensional reduction plot
EMBL-EBI: European molecular biology laboratory – European bioinformatics institute
GMQE: Global model quality estimate
H84/115: Histidine in position 84 and/or 115
H84T: Histidine to threonine in position 84
H115T: Histidine to threonine in position 115
hACE2: Human angiotensin-converting enzyme 2
HIV: Human immunodeficiency virus
HSV: Herpes simplex virus
MEGA: Molecular evolutionary genetics analysis
MSA: Multiple sequence alignment
MDS: Molecular dynamics simulations
MERS-CoV: Middle East respiratory syndrome coronavirus
MM-PBSA: Molecular mechanics Poisson-Boltzmann surface area
NaCl: Sodium chloride
NCBI: National Center for Biotechnology Information
NPT: Constant number (N), pressure (P), and temperature (T)
NVT: Constant number (N), volume (V), and temperature (T)
PRODIGY: Protein binding energy
QMEAN: Qualitative model energy analysis
RBD: Receptor-binding domain
RCSB: Research Collaboratory for Structural Bioinformatics
Rg: Radius of gyration
RMSD: Root-mean-square deviation
RMSF: Root-mean-square fluctuation
RNA: Ribonucleic acid
SARS-CoV-2: Severe acute respiratory syndrome coronavirus 2
T84/115: Threonine in position 84 and/or 115
VOC: Variant of concern
WT: Wild-type
Y83/114: Tyrosine in position 83 and/or 114
YASARA: Yet another scientific artificial reality application
